# Collectanea Medica: Consisting of Anecdotes, Facts, Extracts, Illustrations, &c.

**Published:** 1820-02

**Authors:** 


### COLLECTANEA MEDICA:

CONSISTING OF

### ANECDOTES, FACTS, EXTRACTS, ILLUSTRATIONS, &c.

***Relating to the History or the Art of Medicine, and the Auxiliary Sciences.***

Floriferis ut apes in saltibus omnia libant,
Omnia nos itidem depascimur aurea dicta.

*On the Elephantiasis, as it appears in Hindostan.* By James Robinson, Esq. *Superintendent of the Insane Hospital at Calcutta.* From vol. x. of the *Medico-chirurgical Transactions.*

THE Elephantiasis of the Greeks, or Lepra of the Arabians, is one of the most common diseases of Hindostan, where, however, it is much misunderstood, as two distinct varieties, if not different diseases, indiscriminately bear one name, and, what is worse, are treated alike, though they require very opposite remedies. One variety exhibits the following symptoms:—one or two circumscribed patches appear upon the skin (generally the feet or hands, but sometimes the trunk or face) rather lighter coloured than the neighbouring skin, neither raised nor depressed, shining and wrinkled, the furrows not coinciding with the lines of the contiguous sound cuticle. The skin thus circumscribed is so entirely insensible, that you may with hot irons burn to the muscle before the patient feels any pain. These patches spread slowly until the skin of the whole of the legs, arms, and gradually often the whole body becomes alike devoid of sense: wherever it is so affected there is no perspiration, no itching, no pain, and very seldom any swelling. Until this singular apathy has occupied the greater part of the skin, it may rather be considered as a blemish than a disease; nevertheless, it is most important to mark well these appearances, for they are invariably the commencement of one of the most gigantic and incurable diseases with which the human race is afflicted; and it is in this state chiefly (though not exclusively) that we are most able to be the means of cure. The next symptoms (which occur in some patients at two months, but in others not till after five or six years) are the first which denote internal disease or derangement of any functions. The pulse becomes very slow, from fifty to sixty, not small but heavy, "as if moving through mud;" the bowels are very costive, the toes and fingers numbed, as with frost, glazed and rather swelled, and nearly inflexible. The mind is at this time sluggish and slow in apprehension, and the patient appears always half asleep. The soles of the feet and the palms of the hands then crack into fissures dry and hard as the parched soil of the country, the extremities of the toes and fingers under the nails are encrusted with a furaceous substance, and the nails are gradually lifted up, until absorption and ulceration occur. Still there is little or no pain; the legs and fore arms swell, and the skin is every where cracked and rough. Cotemporary with the last symptoms, or very soon afterwards, ulcers appear at the inside of the joints of the toes and fingers, directly under the last joint of the metatarsal or metacarpal bones, or they corrode the thick sole under the joint of the os calcis or os cuboides. There is no previous tumor, suppuration or pain, but apparently a simple absorption of the integuments, which slough off in successive layers of half an inch in diameter. A sanious discharge comes on; the muscle, pale and flabby, is in turn destroyed; and the joint being penetrated as by an auger, the extremity droops, and at length falls a victim to this cruel, tardy, but certain poison. The wound then heals, and other joints are attacked in succession, whilst every revolving year bears with it a trophy of this slow march of death. Thus are the limbs deprived one by one of their extremities, till at last they become altogether useless. Even now death comes not to the relief of, nor is it desired by, the patient, who, "dying by inches," and a spectacle of horror to all besides, still cherishes fondly the spark of life remaining, and eats voraciously all he can procure: he will often crawl about with little but his trunk remaining, until old age comes on, and at last he is carried off by diarrhœa or dysentery, which the enfeebled constitution has no stamina to resist. Throughout the progress of this creeping, but inveterate complaint, the health is not much disturbed, the food is eaten with appetite, and properly, though slowly, digested; a sleepy inertness overpowers every faculty, and seems to benumb, almost annihilate, every passion as well of the soul as of the body, leaving only sufficient sense and activity to crawl through the routine of existence. This I consider as a distinct variety of Elephantiasis, and from its most striking symptom would name Elephantiasis Anaisthetos. It is probably the "baras" of Avicenna, and is, at its commencement, called in India, sooubharry. I have never seen the larger joints attacked, the nose destroyed, or any bones affected, save those of the hands and feet. The tuberculated species of Elephantiasis which I shall hereafter describe, sometimes supervenes, but is by no means connected with, caused by, or necessarily subsequent to this disease.

For the cure of this affliction our attention is first called to the state of the skin, which is the primary affections, and if we are called in during the first stage, we may always give a favourable prognosis. I have tried almost every thing that has formerly been recommended, and very largely, but in vain; blood-letting, mercury, antimony, are singly of no use, but a happy combination of the two last with a medicine about to be described, applying at the same time topical stimulants, will generally succeed; indeed I have frequently known the sensibility restored entirely, and the disease thereby checked, by the application of a blister to the part, and keeping it open for a few days. Whenever the foot or hand alone is affected, I usually apply a strip of blistering plaister one inch and a half wide all round the limb, just upon the line which marks the sound, from the affected, parts. Where this is inapplicable from the extent of the disease, I apply a solution of muriate of mercury, made as follows:—℞. Hydr. muriat: gr. viij. Acid. muriat: gtt. xx. tere in vit. mort. deinde adde spt: vin. rectif:

℥ss. Aq. fontan. ℔. ij. M. This must be rubbed well upon the skin wherever affected. I, at the same time, give internally as follows: viz. for an adult, ℞ Calomelan. gr. ss. Pulv. antim. gr. iij. ℞ Pulv. cort. radicis asclepiæ giganteæ gr. vj. (ad x.) ter die. This last medicine, which is not in our Pharmacopœias, requires some description. It was communicated to me in 1812, by a Mr. Halked, as a discovery of Mr. Playfair, who afterwards himself favoured me with an account of it. I had hoped he would have given this to the world before now, but as he has not, I cannot refrain from here expressing how much I consider the profession and the world at large indebted to him for the discovery of the most valuable medicine hitherto derived from the vegetable kingdom. Mr. Playfair emphatically describes it as a "vegetable mercury" specific in the cure of lues venerea, leprosy and cutaneous eruptions in general, "the most powerful alterative hitherto known," and an excellent "deobstruent," and thus proceeds:—"In all affections of the skin; I have found it very effectual, and in the jugaru or leprosy of the joints, I have never failed to heal up all the ulcers, and often have produced a perfect cure." In this last complaint, which, until the three last years, I considered leprosy, but which I now believe to be a species of Elephantiasis, I am prepared to agree with Mr. Playfair most fully as to the virtues of the asclepias, called in Hindostan, mudar. I can also bear witness to its powerful effects as a deobstruent and sudorific in almost all cutaneous eruptions, arising from obstructed perspiration and an apathy of the extreme vessels. Its action is quick and decided, causing a sense of heat in the stomach, which rapidly pervades every part of the system, and produces a titillating feel upon the skin from the renewed circulation through the minute vessels. It does not appear to be useful or indeed admissible, where the affection is inflammatory, or the eruption pustular. The great and rapid determination it causes to the skin has an obvious tendency to increase such diseases. I have tried it freely in lues venerea, but cannot venture to recommend it as a substitute for mercury; it will enable you to heal a chancre, but does not eradicate the poison. In the secondary symptoms, however, it is an admirable ally, superseding by its certain efficacy the exhibition of mezereon, sarsaparilla and other vegetables of doubtful utility. Where mercury has been used, but cannot be pushed safely any further, the mudar rapidly recruits the constitution, heals the ulcers, removes the blotches from the skin, and perfects the cure. The only part of the plant useful in medicine is the bark of the roots; it should be gathered in the months of March, April, and May. The bark stripped from the root being well dried, is readily beaten into a fine powder, of which the dose is from three to ten grains thrice a-day for an adult; six grains is enough to commence with. As the plant grows wild everywhere throughout Hindostan, it may be applied advantageously externally. I have often used a poultice made of equal parts of this powder and linseed dust, with decided benefit in bad ulcers, from whatever cause, and even in gangrene; it acts as a detergent in cleansing the sore, and powerfully stimulates the healthy granulations. Decoctions may also be employed where the stomach would reject it in substance. When it causes pain in the stomach, a few grains of magnesia or prepared kali added to each dose will prevent that effect. That this medicine is really the principal in the cure I have no doubt, for I scarcely ever succeeded by any means in curing or even checking the disease before I employed it, and have scarcely ever failed of doing both since.

The second species of Elephantiasis I would denominate E. Tuberculata, from the symptom which always accompanies and forms the most striking character of the disease. It usually commences with patches of the cuticle of the face becoming more florid, and, as Dr. Adams admirably expresses it, "appearing as if semitransparent, splendid, as if the surface were smeared with oil." It then gradually becomes thickened in different parts irregularly, giving a bloated and disgusting appearance to the visage. The alæ of the nose are usually first attacked, then the integuments of the cheeks, temples, and lips, and lastly, the ears, which I have often seen thrice the natural size, nor have I ever seen a case in which they were not affected. The disease in this stage sometimes affects the skin of the arms, hands, neck, and chest, afterwards of the lower extremities and the lower part of the trunk. The skin is not insensible to the touch, but, on the contrary, there is generally a sense of heat and great itching in the tuberculated parts, which are circumscribed, but most irregular; this symptom is followed after a long time by frequent headaches, a great sense of oppression and weight in the head, and a lethargic state of the mind; the tubercles sometimes grow so large and are so numerous as to occupy almost the whole of the face and head, and, from their oily splendour, they produce a most hideous effect; the pulse is slow and feeble, the voice becomes hoarse, and the uvula is generally absorbed without any perceptible ulceration; the vomer is then destroyed, the nose flattened, and, after many years, the bones of the palate and nose become carious. The disease now assumes the appearance of, and is often mistaken for, the secondary symptoms of lues venerea. It is in this species only of Elephantiasis, and in this only at its commencement, that the venereal passion is excessive, probably occasioned by the stimulating irritation of the skin; in the after stages the testicles are absorbed, and if the disease comes on before, it often prevents puberty. The fingers and toes do not (at least till a very late period) become at all numb, or in any way useless, except from the swelling of the skin. When in exertion the skin appears loose upon the body, and shakes on the parts affected as it does in anasarca. From this flabby fulness of the integument, the cheeks hang down, the wrinkles of course deepen, and the patient looks very much older than he is. This disease, like its relation, is slow and very long in progress. I have at this moment a boatman in my budgerow, who has had many tubercles all over his body; for above a year they increased, but, except the irritation they occasion, are productive of no diminution of his strength or health; he works as well and as long as the others, bears exposure to the sun as well, and is not apparently weaker; when he is rowing (which they always do standing) the loose flabbiness of the skin where affected is particularly striking; the skin has lost none of its sensibility, but, on the contrary, is itching constantly; his desires for venery *are more ardent* than before; his hair has not fallen off his eyebrows, chin, or pubes; his ears are twice as big as formerly, the alæ of the nose are swelled, the nostrils dilated, lips tumid, and voice hoarse, obscure, and rather cracked, as if arriving at puberty, though he is thirty years of age. Dr. Bateman gives the most accurate account of Elephantiasis of any author I have read; and yet candidly says he has seen only two cases. He has separated as much as possible the confusion of writers; but still has necessarily mixed the two varieties, which, in this country at least, we find distinct.

The dropping of the extremities, and insensibility of the skin, belong to the first, the tubercles of the skin, ulcerations of the palate, and affection of the cartilages and bones of the face, to the second, variety. In the first, the venereal passion is unaltered, and I have seen many healthy families of children spring from fathers with scarcely a finger or toe remaining: the hair does not drop off from these. In the second variety of Elephantiasis, the mudar *does harm*, and is inadmissible. Arsenic in small doses is the most useful remedy I have yet found; but the certain cure is the grand desideratum. This is equal to the first in longevity; like that, it does not affect the health for many years, and unless it attack the bones of the face and palate, it does not affect life, nor does it produce the same stupefying effect upon the mind or body. Occasionally, you see the patient overwhelmed with both at once, but this is an exception to the rule. I have described the last species less minutely, because it is so well laid down by Dr. Bateman, with the exceptions I have stated. One peculiarity which Dr. Bateman has not mentioned, and which I have generally met with in the second variety, is an oblong glandular swelling in the groin, exactly in the course of the vessels; this is almost always present when the testes are wasting. The hair does not necessarily fall off the pubes, nor does it for many years leave the eyebrows or chin of adults, though the disease prevents its growth when puberty has not taken place. In the *first* variety, I consider mudar, which aggravates the *second*, as the sole effectual remedy.

---

*Speculations on the Cause of the formation of new Vessels in recent Wounds.* From Dr. Carson's Work on the Motion of the Blood.

In the last Number of this journal we gave Dr. Carson's account of the circulation of the blood, as a demonstration: we now adduce an extract from the speculative part of his interesting and ingenious work, on one of the numerous subjects which the doctrine of the automatic motion or active expansion of the ventricles of the heart will probably furnish some important illustrations. After stating the doctrine of John Hunter on the formation of new vessels in recent wounds, he says,

"But it would not, upon this reasoning, be sufficient to allow the mass of blood the wonderful and improbable properties here ascribed to it. It must be supposed to possess others still more singular. It must have judgment to fix the proper direction of the vessels, which it has formed from itself. For it has been often remarked, that a vessel, finding upon the surface immediately opposed to it no vessel with which it can form a communication, goes in quest of one; and it is at a distance frequently that it finds the object of its search. In fact, the new vessels which form the communication between the mouths of vessels opening on divided surfaces are observed to pursue an oblique, crooked, or even a circular, course. Before the time of Mr. Hunter, it was supposed that the arteries in these cases became elongated, and that it was by the stretching of the old, not by the formation of new, vessels, that the vascular communication between divided surfaces was restored. But this is contrary to observation; the vessel which unites the mouths of two opposite vessels, Mr. Hunter ascertained, from an examination of the process in its various stages, to be a new formation. Besides, the same difficulty occurs upon this supposition respecting the direction of the elongated vessel, and the faculty of being able to judge upon the course that is to be taken, would be as necessary to the elongating vessel, as, upon Mr. Hunter's supposition, it is to the blood itself.

"Let us attentively consider the means by which the blood is circulated, and examine whether a more simple and satisfactory explication of these phenomena may not be found.

"When a wound is made in the body, and the parts brought together and kept in that state, the plastic nature of the blood that had oozed out of the arteries, drying near the surface, unites the edges of the wound; but the blood oozing out of the arteries below the surface, remains liquid. The veins in the meantime have become emptied, in consequence of the blood which they contained having taken the less resisted course and returned to the heart. The blood then, which had flowed from the mouths of the arteries into the interstices between the divided surfaces, being less resisted towards the mouths of the veins than in any other direction, necessarily enters the veins, and continues its course to the heart. Other blood follows; and thus a communication is established between the artery and the opposite vein. The blood forming this slow current coagulates upon the external surface, and, in due time, a vessel is thus formed between an artery on one side, and a vein on the other side, of the wound.

"Thus the formation of new vessels communicating between the arteries on one side, and the veins on the other side, of a wound, with a direction conformable to the relative position of the mouths of these vessels, is easily explained upon this simple principle,—the tendency of a fluid to restore the equilibrium of pressure between the particles which compose it; and without being under the necessity of ascribing to the blood properties and even faculties which it is impossible to conceive that it possesses.

"But it may be urged that this explanation will not apply to all the cases. In this instance, there are on each side arteries and veins, the communication between which and the heart has not been interrupted. But in the instances of the transplanted tooth, of the ingrafted spur, and of the transposed flesh, the communication with the heart on one side is completely cut off.

"In reply to this objection, it is contended that, in the case of the tooth, the cavities of the blood-vessels with which it is supplied, from the incompressible nature of the substance of the tooth, are always of the same dimensions. If, therefore, a diminution of pressure, which was previously equal, be made at one end of a small incompressible tube, and, if the other end of the tube be inserted in a liquid, a current will necessarily be produced from one end of the tube to the other. This canal may be supposed to perform the function of a vein, and its demands, as they are perpetually renewed, will be supplied by an artery on the other side of the tooth; the same will take place in all the vessels of the tooth; so that a communication will be established between the tooth and the parts which surround it, the circulation will be restored, and the life of the part preserved. The same reasoning may be trsnsferred to the transplanted spur of the cock.

"But it may still be urged, that this reasoning is not applicable to a piece of flesh which has been inserted, as the vessels belonging to it are compressible, and their tubular form not so easily preserved. It is to be observed in this case, that though the larger veins collapse upon abstraction from the body, yet the capillary veins, though possessed of the same structure, may, in the same situation, be supposed to preserve their tubularity in a considerable degree, in consequence of the minuteness of the circles formed by their composing fibres, and the great curvature and proximity of the tubular arches. As the transverse section of the larger arteries forms a complete circle, the minute arteries may be admitted to preserve their tubularity in a still more perfect degree. The blood which is poured from the arteries, the connexion of which with the heart has not been interrupted, into the interstices between the approximated and adherent surfaces, will in time accumulate and be compressed by the resistance opposed by the two surfaces.

"The transplanting of glandular bodies, as of the testes of animals, may be still more certainly effected, as the vessels of these parts preserve their tubularity more perfect, and continue more permeable, after their separation from the body.

"Hence, from the conjoined operation of the pressure affecting the extravasated blood, and of the exhausting influence extending to it from the heart through the veins, in connexion with the capacity which the small vessels possess of preserving their tubularity, and of the adherence of the principle of life to the newly applied part, the circulation between that part and the adjoining substance will be gradually established, their union effected, and the life of the former preserved.

"Thus, from the principles which have been attempted to be established in the preceding pages, there is derived an easy, and I hope, satisfactory, explanation of the manner in which the parts of a body that have been divided are brought to unite; and a step seems to have been made towards ascertaining the process which nature pursues in the greatest of all her works,—the formation of animals."

